# No evidence that gaze anxiety predicts gaze avoidance behavior during face-to-face social interaction

**DOI:** 10.1038/s41598-022-25189-z

**Published:** 2022-12-09

**Authors:** Daniel Tönsing, Bastian Schiller, Antonia Vehlen, Ines Spenthof, Gregor Domes, Markus Heinrichs

**Affiliations:** 1 Laboratory for Biological Psychology, Clinical Psychology and Psychotherapy, Department of Psychology, Albert-Ludwigs University of Freiburg, Stefan-Meier-Straße 8, 79104 Freiburg, Germany; 2 Freiburg Brain Imaging Center, University Medical Center, Albert-Ludwigs University of Freiburg, Freiburg, Germany; 3 Department of Biological and Clinical Psychology, University of Trier, Trier, Germany

**Keywords:** Psychology, Human behaviour, Anxiety

## Abstract

Eye contact is an indispensable social signal, yet for some individuals it is also a source of discomfort they fear and avoid. However, it is still unknown whether gaze anxiety actually produces avoidant gaze behavior in naturalistic, face-to-face interactions. Here, we relied on a novel dual eye-tracking setup that allows us to assess interactive gaze behavior. To investigate the effect of gaze anxiety on gaze behavior, we a priori created groups of participants reporting high or low levels of gaze anxiety. These participants (n = 51) then performed a semi-standardized interaction with a previously unknown individual reporting a medium level of gaze anxiety. The gaze behavior of both groups did not differ in either classical one-way, eye-tracking parameters (e.g. unilateral eye gaze), or interactive, two-way ones (e.g. mutual gaze). Furthermore, the subjective ratings of both participants’ interaction did not differ between groups. Gaze anxious individuals seem to exhibit normal gaze behavior which does not hamper the perceived quality of interactions in a naturalistic face-to-face setup. Our findings point to the existence of cognitive distortions in gaze anxious individuals whose exterior behavior might be less affected than feared by their interior anxiety.

## Introduction

Paying visual attention to social stimuli forms the basis of our social interactions: Attention to the faces and eyes can reveal a myriad of socially relevant information comprising the partners’ emotional and mental state^1,2^. In particular, direct eye contact is known to facilitate facial emotion recognition^3^, to activate neural regions associated with social processing^4^, and to promote neural coherence between two interacting partners^5^. Yet for socially anxious individuals, social signals like eye contact can also represent feared cues of scrutiny and negative social evaluation resulting in gaze anxiety^6^. Findings on whether gaze anxiety actually results in gaze avoidant behavior (i.e. avoiding direct eye contact), however, are equivocal^7–9^. The situation is further complicated by the fact that results from laboratory research studying the association between anxiety and gaze behavior using “face-to-screen” paradigms (e.g. passively viewing faces or videos, interacting with others via webcams)^10^ might not be transferable to actual social interactions occurring “face-to-face”. Therefore, this study was designed to investigate the effects of gaze anxiety on gaze behavior directed towards the eyes/face applying a dual eye-tracking setup that enables the assessment of interactive gaze behavior.

While gaze anxiety is associated with several clinical conditions that involve dysfunctional social interactions, clinicians and researchers usually discuss it as a hallmark feature of social anxiety disorder (SAD). This disorder is highly prevalent and disabling^11,12^ and characterized by both intense fear and avoidance of social situations accompanied by potential negative evaluations by others^13^. As faces in general and eyes in particular convey cues of scrutiny, patients with SAD are supposed to fear eye contact. Indeed, research using factor analyses reveals that the single item “fear of eye contact” accounts for most of the variance in the factor distinguishing healthy controls from SAD patients^14,15^. In line with this, SAD patients exhibit greater activation in arousal-related brain regions during direct eye gaze compared to averted gaze^16^. Moreover, individuals suffering from more severe social anxiety also report greater gaze anxiety in studies involving clinical and nonclinical samples^17–20^. In sum, gaze anxiety seems to play a prominent role in social anxiety. But does gaze anxiety actually result in gaze avoidance?

Empirical evidence demonstrating that socially anxious individuals actually exhibit gaze avoidance is far more equivocal than expected^7–9^. On the one hand, and in line with clinical observations^21,22^ and SAD patients’ self-reports^23^, research has shown that individuals with sub-clinically and clinically relevant social anxiety levels display gaze patterns consistent with avoiding gaze^6,24^. On the other hand, however, there is also evidence of normal or increased eye gaze in socially anxious individuals^25^. In particular, the few studies investigating gaze behavior in actual, live social interactions have yielded inconsistent findings^26–30^ with several reporting similar gaze behavior in individuals with high and low levels of social anxiety^24,31^. This has led some researchers to suppose that when interacting socially, individuals with high gaze anxiety might suffer from cognitive distortions regarding their own gaze behavior and might be behaviorally compensating for their anxiety so as to demonstrate gaze behavior corresponding with social norms^31,32^. Altogether, we still have no definite answer to the question whether gaze anxiety leads to gaze avoidance. More specifically, research on gaze behavior in naturalistic face-to-face social interactions is needed, as these situations are most frequent in and affect an individual’s everyday life.

To track two interacting participants’ gaze behavior simultaneously with no devices interfering with natural eye contact, we chose a novel, recently validated remote eye-tracking setup from our laboratory^33^ that can be used for a naturalistic dual eye-tracking experiment. This setup consists of a natural face-to-face conversation while ensuring eye-tracking accuracy within an experimentally controlled, laboratory setting. Investigating gaze behavior in a naturalistic environment is important, because gaze behavior in daily social interactions differs from gaze behavior in computer facial-viewing tasks in several fundamental characteristics^34–36^. First, the mere physical presence of another individual induces a social context that activates social norms affecting gaze behavior. For example, participants might feel more obliged to hold eye contact from face-to-face in order to avoid negative consequences (e.g. negative evaluation), no matter how pleasant or unpleasant that might be^36–39^. In contrast, in computer facial-viewing tasks or interactions done from “face-to-screen” (e.g. via webcam) participants might not sense this social pressure to the same degree and succumb to this matter by avoiding eye contact. Second, interacting individuals mutually communicate with each other via their gaze behavior, as we both “listen” and “speak” using our eyes^28,36,40^. In particular, mutual eye gaze is a crucial state of social communication modulating the attention of two interacting individuals^41^. Notably, the eye tracking setup we applied provides unique information on this variable by making data from simultaneously tracked gaze behavior available.

With these considerations in mind, we set out to study whether self-reported gaze anxiety is associated with gaze behavior in naturalistic, face-to-face social interactions. For that purpose, we a priori created groups of participants reporting either high or low levels of gaze anxiety using the Gaze Anxiety Rating Scale^20,23^. To generate comparable experimental conditions across participating groups, participants then performed a semi-structured interaction (“fast-friends procedure”^42^) with a previously unknown individual reporting a medium level of gaze anxiety. This procedure aims to mimic a typical daily rapport-building social interaction with an unknown stranger through escalating, mutual self-disclosure in a series of turn-taking questions^42,43^. Furthermore, it enables us to compare gaze behavior between talking and listening situations. This could be relevant, as cognitive demands are supposedly higher while talking—a situation potentially associated with more likely gaze avoidance in gaze-anxious participants^28,44^. Analysing both classical one-way (e.g. unilateral eye gaze; for details see Methods), as well as interactive two-way eye-tracking parameters (e.g. mutual eye gaze; for details see Methods), we thus compared gaze behavior between participants with high and low levels of gaze anxiety. We also analyzed potential group differences with regard to subjective ratings of the interaction quality. Expanding upon previous research by investigating gaze behavior in a naturalistic, face-to-face interaction, we aimed to shed light on whether gaze anxiety is associated with gaze avoidance as predicted by several theoretical models. Alternatively, the naturalistic face-to-face environment might strongly activate social norms to engage in eye contact, which in turn could dilute any effects of gaze anxiety on gaze behavior.

## Methods

### Participants

One hundred and twenty participants were recruited via on-campus and online announcements at the University of Freiburg. To minimize the risk of self-selection, general recruitment took place without reference to the actual study purpose and with a cover story (“how do we communicate in a digital world?”) and cover questionnaires. Participants completed an online screening to monitor the exclusion criteria (visual impairments exceeding 3.0 dioptres, current eye infections, allergic reaction and irritated eyes, any other type of prescriptive visual aid, any mental health issues, alcohol or drug abuse and psychiatric or therapeutic treatment in the past two years). To avoid the cofounding effects of mating, we focussed on participants with a heterosexual orientation^45^. Furthermore, participants in this screening completed the Gaze Anxiety Rating Scale^23^, which we used to a priori create groups of participants reporting either high, medium, or low levels of gaze anxiety (for details, see Procedure). On the basis of this a priori selection of two extreme groups, we assumed a large effect size in performing the power analysis (f = 0.40, α = 0.05, β = 0.80, number of groups = 2, ANOVA fixed effects, omnibus, one-way) which resulted in an optimal sample size of 52 participants. To account for potential dropouts, we aimed at a sample size of 60 participants of interest and 120 participants in total (including the interaction partner reporting a medium level of gaze anxiety). Because of technical problems while recording eye-tracking in one of the two interacting participants, data from nine interaction pairs (i.e. 18 participants) had to be excluded. Consequently, our total sample comprised 102 participants (54 female, 48 male) aged *M* = 23.54 years (*SD* = 3.38) that all matched our eye -racking data quality restrictions (for more details see Table 1). Note that our analyses focus on comparing participants reporting high or low levels of gaze anxiety, comprising a total number of 51 participants of interest (for detailed descriptive parameters of groups, see Table S1). The University of Freiburg Ethics Committee approved this study whose methods were carried out in accordance with relevant guidelines and regulations. All participants gave written informed consent before the experiment took place and received 15.00 € for participating.

**Table 1:** Accuracy, precision and robustness for the calibration validations *wall*, *face (pre)* and *face (post)*.

	Wall		Face (pre)		Face (post)		
	*M*	*SD*	*M*	*SD*	*M*		*SD*
Accuracy	0.44°	0.20°	0.56°	0.20°	0.64°		0.27°
Precision (SD)	0.25°	0.08°	0.26°	0.08°	0.27°		0.09°
Robustness (%)	98.64	2.10	99.01	1.97	98.73		2.87

Mean (*M*) and standard deviation (*SD*) for eye-tracking quality are shown for all three points of measurements (wall, face pre, face post). Robustness is reported in percent, accuracy and precision in visual angle. Precision (SD): precision calculated as standard deviation of the data sample. Of the 1734 trails, 34 markers (1.96%) had to be corrected because of saccades. 70 markers (4.04%) had to be postponed due to suboptimal marker time setting. 9 trails (0.52%) had to be excluded due to experimenter error. 13 trails (0.75%) had to be excluded because of robustness < 80.00%.

### Setup

**Figure 1:**
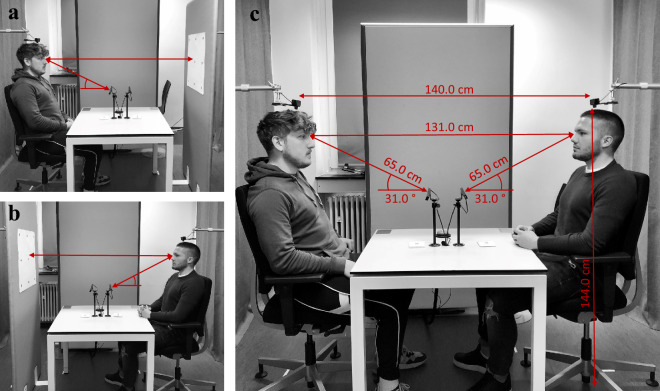
Dual eye-tracking calibration procedure and setup. Shown are experimental setups and relevant distances during the calibration procedure for participant A (**a**), participant B (**b**), and the face-to-face interaction between both participants (**c**). Distances are given in cm and angles are given in degree. Participant A (randomly picked) was taken to the experimental room and seated on a chair facing a calibration wall. The chair was moved and adjusted to ensure the participant’s head was close to the camera and at the described distances to the eye-tracker and camera (**a**). Participant A was introduced to first find a comfortable seating position. Because we adjusted the chair’s height, a participant’s eyes were on the same level regardless of their height. Participant A ran through a 9-point-calibration procedure. After participant A’s calibration was completed, a calibration validation using the calibration wall was conducted. Participant A was instructed to gaze at a randomized order of calibration points. The chair’s position and settings were then marked, and participants A left the room. Participants A’s chair was removed and replaced by the calibration wall. On the other side, the calibration wall was replaced by the second chair. Participant B entered the room and was seated on the second chair (**b**). The same calibration and validation procedure was conducted for participant B. Then participant B stayed seated, the calibration wall was removed and participant A’s chair was placed on the marked position. Participant A returned to sit on that chair (**c**). For the second validation, participants were asked to gaze at the opponent's facial features (left eye, right eye, mouth, and the nose tip) for 1 s, starting in random order. This validation procedure was then repeated after the “fast-friends-procedure”. Note that we obtained informed consent from all displayed subjects for publication of identifying images in an online open-access publication.

The experiment took place in a bright room (size: 3.6 m × 2.3 m, height: 2.6 m) with constant artificial lighting (570 lx; daylight was blocked by window blinds) and a constant temperature. A white table (size: 80 cm × 80 cm; height: 72 cm) and two identical, movable and height-adjustable chairs facing each other were placed in the experimental room. Note that the chairs’ seating height and position were only adjustable with the help of the experimenter, and that one of the chairs was replaced by a movable vertical partition wall (180 × 80 cm) during the calibration procedure (see Fig. 1a,b). On the calibration sheet, there were nine black cross-hairs with two red circles with a total radius of 3.5 cm (1.5° visual angle) on a white background. There were three cross-hairs in three rows, each 20 cm (8.64° visual angle) vertically and horizontally apart from each other, resulting in a calibration area of 40 × 40 cm (17.3° × 17.3° visual angle).

To record eye-tracking data, two Tobii X3-120 remote infrared eye-trackers (sampling frequency: 120 Hz) were positioned on the table facing opposite sites. Two cameras (Logitech C920) were installed above the participants’ heads to record both participants’ fields of view (FHD resolution: 1920 × 1080 px with 30 fps). A movable green push-button was placed in the middle of the table. It was used to synchronize both eye-tracking data streams and to segment the interaction (see Procedure). Furthermore, a manual ringing bell was placed on the table. Figure 1c shows the final setup including all angles and distances.

### Material

#### Gaze Anxiety Rating Scale

The Gaze Anxiety Rating Scale (GARS) assesses subjectively experienced fear and avoidance of eye contact^20,23^. Its items were originally derived from unstructured interviews with SAD patients, comprising 17 situations (e.g. “Giving a speech”, “Speaking to someone you find attractive”, “Expressing a disagreement”) involving the increased fear or avoidance of eye contact these patients reported. For each of these situations, ratings of fear and avoidance are collected on a 4-point Likert scale (0 = none to 3 = severe). Summing up responses to specific items, one then calculates sub scores of GARS-fear and GARS-avoidance, as well as the overall GARS-score (which we used to create participant groups in this study). The GARS and its German translation are known to be very reliable and to constitute a valid self-report measure of fear and avoidance of eye contact^20,23^. In the German version internal consistencies (Cronbach’s α) for the total score and the subscales were very high, ranging from α = 0.90 to α = 0.95. Domes et al.^23^ reported norm indices for the overall GARS-score in a German-speaking population with *M* = 21.1, (*SD* = 15.3, range 0–51), demonstrating that our participant groups revealed extraordinarily low (*M* = 6.58, *SD* = 3.01) and high levels of gaze anxiety (*M* = 39.17, *SD* = 5.65).

#### The fast-friends procedure

The “fast-friends-procedure” (FFP) scaffolds semi-standardized communication and builds rapport among unknown others through mutual self-disclosure in a series of turn-taking questions^42,46,47^. Here, we applied 12 questions from the original FFP translated to German (e.g. “Given the choice of anyone in the world, whom would you want as a dinner guest?”; for details see Table S2).

#### Trait measures

In addition to the GARS, we assessed social anxiety (social anxiety and fear of negative evaluation: Social Interaction Anxiety Scale, SIAS-D^48,49^) and general cognitive ability (verbal intelligence quotient: Wortschatztest, WST^50^).

#### Subjective ratings of interaction quality

To assess an individual’s subjectively experienced interaction quality, we collected responses to 12 items via a 7 point Likert Scale (1 = not at all, 7 = very much). These items were about liking the other person, the general feeling towards the other person, the wish to meet that person again, perceived similarity to the other person, enjoyment of the interaction, laughing during the interaction, fun of the interaction, perceived self-disclosure of the other person, perceived intimacy, perceived honesty, perceived quality of the relationship (compared to one’s own other relationships and compared to relationships of other persons in general). We also used the *Inclusion of Other in the Self Scale* (IOS) to assess perceived interpersonal closeness (participants have to choose one pair of circles from seven with different degrees of overlap ranging from 1 = no overlap to 7 = most overlap)^51^.

### Experimental procedure

Before the laboratory appointment took place, participants completed the described trait measures online. Based on their GARS overall scores and percentile rank (PR), participants were first assigned to one of three groups: GARS low (0.0–20.0 PR, score ≤ 10), GARS medium (GARS medium: 40.0–60.0 PR, score: 18–24) or GARS high (GARS high: 80.0–100.0 PR, score ≥ 32^23^). In the experiment, a participant with either low or high GARS-scores then interacted with a same-sex participant from the average GARS group. The experiment started right after the second participant arrived to make sure participants could not interact before the investigation. We also made sure that participants had never met each other before taking part in the experiment. Participants then provided informed consent and were then given a standardized verbal instruction regarding the experimental procedure. Specifically, they were instructed to participate in a social interaction task in which they would have to pose and answer prepared questions in alternating order. They were also informed that this interaction would be recorded for study purposes, i.e. analyzing verbal and non-verbal behavior during social interactions. Other than these, no specific instructions were given to enable a semi-standardized, naturalistic social interaction between participants.

Afterwards, the eye-tracking and video recordings were prepared. For that purpose, one randomly chosen participant (participant A) was taken to the experimental room where settings (e.g. seating height, chair position) had been optimized for the eye-tracking recording, and the eye-tracking calibration procedure was completed using the calibration wall (for details, see Fig. 1A). During this procedure, the other participant (participant B) waited in another room. After successful calibration, participants switched rooms, and the same procedure was repeated in the experimental room with participant B sitting on the opposite chair (see Fig. 1B). Afterwards, the participant in the waiting room returned to the experimental room where the previously defined experimental settings were restored with both participants now sitting in front of each other (see Fig. 1C). A final validation procedure was then performed with both participants gazing on the interaction partner’s distinct facial features.

The experimenter then left the room, and the social interaction (i.e. FFP) task began. For that purpose, six printed cards with questions 1–6 of the FFP were placed in front of participant A, and the remaining cards with questions 7–12 were placed in front of participant B. Participant A started by reading aloud the first question and then pressing the green button to mark each question in the recordings. Afterwards, both participants (first B, then A) answered that question. This procedure was repeated until the sixth question. The procedure for questions 7–12 was vice versa with participant B posing the questions. When participants were done posing all 12 questions, they were instructed to remain seated and ring a bell placed on the table. The experimenter entered the room again, and a second validation procedure on facial features was performed for both participants (for details, see Fig. 1). For the last part of the experiment, participants were seated in different rooms where they completed the social interaction questionnaire. Finally, participants received their payment.

### Analysis of eye-tracking data

#### Preprocessing

All calculations were based on averaged binocular data. Raw data of the segments were visually inspected for saccades. In cases of a saccade occurring within the segment (e.g. the marker was set before the participant fixated on the announced point), markers were corrected. For more details, also see Vehlen et al.^33^.

#### Robustness, precision, and accuracy

Robustness was calculated as the percentage of valid data for a specific time sequence (for details, see Table 1). Accuracy was calculated as the mean offset of the gaze point position in relation to the target point position and is reported in degrees of gaze angle. Precision as a measure of variance was calculated via the standard deviation (*SD*) of data samples and is reported in degrees of gaze angle. Calculations of basic quality indices were conducted via in-house scripts in Matlab (2018a, version 9.7.0).

#### Definition of areas of interest

First, scene videos were analyzed by the facial landmark detection tool *Open Face*^52^ to generate facial landmarks. Then the limited-radius voronoi tessellation method^53^ with a radius of 2° was applied to receive Areas of Interest (AOI) for the eyes (adding up the right and left eyes), nose, and mouth^54^. Furthermore, we took an ellipse around the face as an additional AOI.

#### Analysis of one-way eye-tracking data

Total dwell time on a specific AOI was defined as the percentage of total frames within a specific segment comprising all gaze behavior (fixations, saccades, etc.). In addition, the percentage of missing data was calculated comprising all frames with no data available due to gaze outside the calibration area or loss of eye tracking. Groups did not differ in this measure, *F*(1, 49) = 0.01, *p* = 0.916, η^2^ < 0.001. For the one-way gaze analysis, we calculated the average dwell time on the opponent’s eyes, separately for speaking phases, listening phases, and aggregated across all phases.

#### Analysis of two-way eye-tracking data

For the two-way gaze analysis, we calculated mutual eye gaze on the opponent’s eyes, separately for speaking phases, listening phases, and aggregated across all phases. Mutual eye gaze was defined as time periods in which both participants gaze at each other’s eyes simultaneously (to reduce noise we focused on events in which both participants gazed at the other’s eyes for at least 200 ms; note that findings did not change when this constraint was not applied).

#### Analysis of speaking and listening phases

For the conversation analysis, two independent raters were trained to judge whether participant A or participant B was reading aloud a question, listening, speaking, or no person was speaking. All conversations were rated by both raters, and interrater-reliability for speaking and listening phases indicated very good agreement across raters (Cohen’s κ = 0.93). To temporally match eye-tracking with video data, a marker was set at the beginning of the recording in all data streams. Subsequent events were then temporally matched in reference to this start marker using in-house scripts in Matlab.

### Statistical analysis

Using univariate ANOVAs, we compared participants of the “GARS low” and the “GARS high” group (between-subjects factor “group”) regarding gaze anxiety (dependent variable “GARS-score”), social anxiety (dependent variable “SIAS score”), age (dependent variable “age”), and cognitive ability (dependent variable “Verbal Intelligence score”). Next, using univariate ANOVAs, we compared participants in the “GARS low” and “GARS high” group (between-subjects factor “group”) in their gaze behavior (one-way analysis: “dwell time on the opponent’s eyes”; two-way analysis: “mutual eye gaze”). We also analyzed differential effects of gaze anxiety on gaze behavior between speaking phases and listening phases, by adding “activity” (speaking vs. listening) as a within-subjects factor to a mixed ANOVA. Following up on this overall ANOVA, we also calculated two post-hoc univariate ANOVAs that focused on gaze behavior displayed during speaking or listening phases. To analyze effects of gaze anxiety on subjective ratings of interaction quality, we calculated a MANOVA (between-subjects factor “group”). To control for any sex effects, we added “sex” as a covariate in the analyses. We also conducted the same analysis using the AOIs “mouth”, “nose”, and “whole face”. All statistical analyses were conducted in statistical software SPSS (version 28.0^55^) with the level of significance set to *p* < 0.05. In cases of heterogeneity of covariance, Greenhouse–Geisser corrections were applied. To confirm the robustness of the reported analyses, we also conducted non-parametrical Wilcoxon tests (although ANOVAs are supposed to be robust against violations of the normality assumption in large samples).

In addition to traditional null-hypothesis significance testing, we performed equivalence testing^56,57^ using the two one-sided tests (TOST) procedure for Welch’s t tests for independent samples^58^. Thereby, we wanted to examine the hypothesis that the difference in gaze behavior participants in the two GARS groups revealed is smaller than what is considered to be a meaningful effect size in this population. Given that we compared the gaze behavior of a priori created groups of participants with extremely low (0–20 percentile) and high (80–100) levels of self-reported gaze anxiety (i.e. GARS-scores), we set the smallest effect size of interest to a large effect, resulting in meaningful effect size bounds of *d* = –0.80 (lower) and *d* = 0.80 (upper).

As non-significant results using frequentist statistics do not mean the alternative hypothesis is true, we performed Bayesian analyses taking the Bayesian t-test approach^59,60^. The null hypothesis postulates that two groups do not differ in gaze behavior *H*_0_: δ = 0. The two-sided alternative hypothesis (*H*_*1*_) states that the GARS high group differs from the GARS low group. The likelihood for the alternative hypothesis given the data (BF_10_), compared to the null hypothesis was assessed using the default prior option provided by JASP. The Bayes Factor can indicate higher likelihood for one hypothesis. Equivalence testing and Bayesian independent sample *t*-test were performed in JASP (Version 0.16.3, 2021^61^).

## Results

### Self-reported gaze anxiety

**Figure 2:**
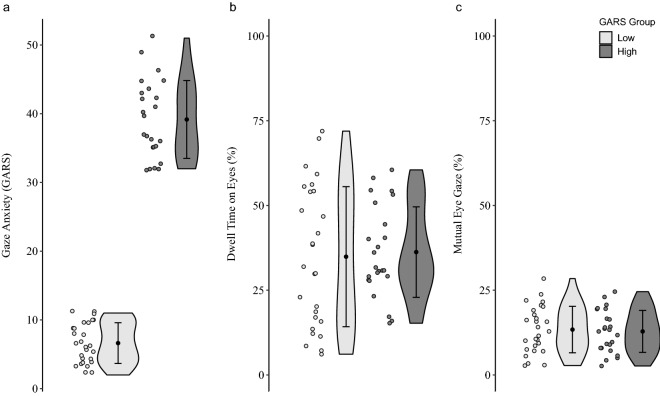
The effect of gaze anxiety on total dwell time on opponent’s eyes and mutual eye gaze for GARS group low and high. (**a**) Gaze Anxiety Rating Scale (GARS), (**b**) total dwell time on eyes (**c**) and mutual eye gaze for both GARS groups low and high. Data distribution is displayed with violin plots including mean (black dot) and standard deviation (brackets) and individual means for each participant. Gaze anxiety is displayed as a sum score, dwell time on eyes and mutual eye gaze as a percentage of total time.

As intended, participants in the “GARS high” group reported significantly more gaze anxiety than those in the “GARS low” group (*F*(1, 49) = 683.99, *p* < 0.001, η^2^ = 0.933; Z = −6.12, *p* < 0.001; see Fig. 2a and Table S1 for descriptive statistics). Participants in the “GARS high” group also displayed significantly higher social anxiety (see *F*(1, 49) = 33.03, *p* < 0.001, η^2^ = 0.403; *Z* = −4.58, *p* < 0.001; see Table S1 for descriptive statistics). Note that the two GARS subscales “GARS-fear” and “GARS-avoidance” correlated closely (*r* = 0.791, *p* < 0.001), suggesting that participants with high gaze anxiety also subjectively perceive and report high gaze avoidance. Note furthermore that these two groups did not differ in age (*F*(1, 49) = 0.66, *p* = 0.422, η^2^ = 0.013; *Z* = −0.53, *p* = 0.596) or cognitive ability (*F*(1, 49) = 3.14, *p* = 0.083, η^2^ = 0.060; *Z* = −1.34, *p* = 0.181).

### Gaze behavior

#### One-way eye-tracking analysis

First, we compared gaze behavior between participants with high and low levels of gaze anxiety, focusing on the participant’s gaze behavior directed towards the eyes/face. For that purpose, we calculated a univariate ANOVA with the dependent variable “dwell time on the opponent’s eyes” and the independent variable “GARS group” (low vs. high). We detected no significant effect of gaze anxiety on gaze behavior (*F*(1, 49) = 0.40, *p* = 0.530, *η*^2^ = 0.008; non-parametric testing: Z = −1.00, *p* = 0.317; see Fig. 2b). Equivalence testing revealed that the effect of gaze anxiety on gaze behavior is statistically equivalent to zero when testing against the presence of a large effect (*T*(44.96) = 2.60, *p* = 0.006). The Bayes Factor (BF_01_ = 3.45) indicated that the data are approximately 3.45 times more likely to occur under *H*_*0*_ than under *H*_*1*_*.*

Secondly, we analyzed whether gaze anxiety specifically affects gaze behavior when participants are either speaking or listening (running the same ANOVA, but this time with the dependent variables “dwell times on the eyes while speaking” or “dwell times on the eyes while listening”). Again, a mixed ANOVA with the dependent variable “dwell times on the eyes” and independent variables “GARS group” (low vs. high) and “activity” (speaking vs. listening) demonstrated no significant effects of gaze anxiety on gaze behavior (main effect “GARS group”: *F*(1, 49) = 0.08, *p* = 0.784, *η*^2^ = 0.002). There was also no evidence indicating significant effects of gaze anxiety on gaze behavior in separate post-hoc ANOVAs and using equivalence testing for listening periods (*F*(1, 49) = 0.63, *p* = 0.430, *η*^2^ = 0.013; non-parametric testing: Z = −0.85, *p* = 0.396*; equivalence testing: T*(48.26) = 2.07, *p* = 0.022) and speaking periods (*F*(1, 49) = 0.11, *p* = 0.741, *η*^2^ = 0.002; non-parametric testing: *Z* = −0.15, *p* = 0.880; equivalence testing: *T*(43.00) = -2.51, *p* = 0.007). Furthermore, a participant’s gaze anxiety did not significantly affect how they modulated their dwell time on eyes while listening rather than speaking (main effect “activity” with higher levels of dwell times on the eyes during listening phases: *F*(1, 49) = 21.39, *p* < 0.001, *η*^2^ = 0.304; interaction effect “GARS group X activity”: *F*(1, 49) = 3.69, *p* = 0.061, *η*^2^ = 0.070). Please note that all our findings were very similar when adding “sex” as a covariate in the analyses and when applying the same analysis to the AOIs “nose”, “mouth”, and “whole face” (for details, see Table S3).

In sum, the analysis of one-way eye-tracking parameters, which does not take into account the opponent’s gaze behavior, provided no evidence that gaze anxiety modulates gaze behavior directed towards the eyes/face in face-to-face interactions.

#### Two-way eye-tracking analysis

Next, we investigated the effects of gaze anxiety on gaze behavior when taking into account both the participant’s and opponent’s gaze behavior by analyzing mutual eye contact by calculating a univariate ANOVA with the dependent variable “mutual eye gaze” and the independent variable “GARS group” (low vs. high). Again, the effect of gaze anxiety on gaze behavior was not significant (*F*(1, 49) = 0.03, *p* = 0.873, *η*^2^ = 0.001; non-parametric testing: *Z* = −0.56, *p* = 0.577; see Fig. 2c), and equivalence testing revealed that the effect of gaze anxiety on gaze behavior is statistically equivalent to zero when testing against the presence of a large effect (*T*(48.47) = 2.71, *p* = 0.005). The Bayes Factor (BF_01_ = 3.43) indicated that the data are approximately 3.43 times more likely to occur under *H*_*0*_ than under *H*_*1*_*.*

As in the one-way analysis, we then tested for gaze-anxiety effects on gaze behavior running the same analyses, but with the dependent variables “mutual eye gaze while speaking” or “mutual eye gaze while listening”. Again, gaze anxiety did not modulate gaze behavior (overall main effect “GARS group”: *F*(1, 49) = 0.01, *p* = 0.907 *η*^2^ = 0.000; speaking: *F*(1, 49) = 0.01, *p* = 0.940, *η*^2^ = 0.000; non-parametric testing: *Z* = -0.51, *p* = 0.610; equivalence testing: *T*(46.46) = -2.80, *p* = 0.004; listening: *F*(1, 49) = 0.09, *p* = 0.768, *η*^2^ = 0.002; non-parametric testing: *Z* = -0.17, *p* = 0.865; equivalence testing: *T*(47.27) = 2.55, *p* = 0.007). In contrast to the one-way analyses, we observed no differences in mutual eye gaze between speaking and listening periods (referring to the participant’s activity; main effect “activity”: *F*(1, 49) = 0.62, p = 0.434, η2 = 0.013; interaction effect “GARS group X activity”: *F*(1, 49) = 0.12, *p* = 0.733, η^2^ = 0.002). Again, all findings were very similar when adding “sex” as a covariate in the analyses and when using “mutual face gaze” as dependent variable (for details, see Table S4).

In sum, analyzing two-way eye-tracking parameters corroborated the assumption that there is no evidence of gaze anxiety modulating gaze behavior directed towards the eyes/face in face-to-face interactions. Furthermore, correlation analyses revealed no systematic relation between GARS total score, subscales, SIAS and any one-way and two-way gaze data parameters (for more details see Tables S5, S6, S7).

### Subjective ratings of interaction quality

In the last analysis, we tested whether self-reported trait gaze anxiety would have any effects on subjective ratings of interaction quality. First, we compared the responses of participants between the “high GARS” and “low GARS” group on the 12 items (see Methods) and the IOS by calculating a MANOVA. Both the multivariate effect of “GARS group” (*F*(13, 37) = 0.33, *p* = 0.983, *η*^2^ = 0.103) as well as univariate effect of “GARS group” regarding single items (all *F*(1, 49) < 1.98; all *p*s > 0.166) were non-significant. This finding demonstrates that despite the strong group difference in a priori reported gaze anxiety (*η*^*2*^ = 0.933), participants in both groups perceived the interaction quality similarly after the experiment (also see Table S8).

It is still however possible that interaction partners noticed an effect of a participant’s gaze anxiety. We therefore ran the same analyses on the interaction partners’ subjective ratings. Again, no significant effect of “GARS group” appeared (multivariate effect: *F*(13, 37) = 0.36, *p* = 0.974, *η*^2^ = 0.113; univariate effects: all *F*(1, 49) < 1.32, all *p*s > 0.256), demonstrating that interaction partners’ perceptions of the interaction quality were not influenced either by a participant’s gaze anxiety (also see Table S9).

## Discussion

In the present study, we investigated the impact of self-reported gaze anxiety on actual gaze behavior directed towards the eyes/face in dyadic communication. Two groups of participants with either high or low levels of gaze anxiety conducted a semi-structured, naturalistic face-to-face interaction (i.e. fast-friends procedure) with a previously unknown participant with a medium level of gaze anxiety. Capitalizing on a novel dual eye-tracking setup, we analyzed gaze behavior with regard to both one-way (unilateral eye gaze) and interactive, two-way (mutual eye gaze) eye-tracking parameters. Furthermore, in our analyses, we differentiated the gaze behavior displayed during speaking or listening situations, which are associated with varying levels of cognitive demands. We found no evidence of significant differences between participants with low and high gaze anxiety. Likewise, we detected no evidence that a participant’s gaze anxiety affected their quality of social interaction, as subjective ratings of the interaction immediately after the experiment by both participants and their interaction partners were similarly positive. Furthermore, equivalence testing allowed to us to exclude differences of large effect sizes, and Bayesian analyses yielded moderate evidence^62^ of absent group differences. While even gaze anxiety’s subtle effects on gaze behavior might still be highly consequential in social interactions, our findings indicate overall a pronounced difference in self-reported gaze anxiety and actual gaze avoidance.

This finding contributes to the ongoing debate on the genuine relationship between gaze anxiety and gaze behavior, and on the role that gaze anxiety plays in social anxiety more generally. Many prominent clinical models claim that gaze anxiety causes gaze avoidance and thereby significantly aggravates worries about being negatively evaluated by others^31,32^. Meanwhile, there are ideas that gaze-anxious individuals are cognitively biased when evaluating and perceiving their own gaze behavior, and might indeed exhibit normal gaze behavior^31,32^. Past empirical research reported heterogeneous evidence (for reviews see^7,8^). This could also be due to the fact that so far, only recent pioneering eye-tracking studies have studied the relationship between gaze anxiety and gaze behavior in naturalistic face-to-face interactions^26,63^, probably because such setups are technically and analytically complex. Here, we applied a new dual eye-tracking setup recently validated in our laboratories^33^, which enabled us to study gaze behavior in a face-to-face, live interaction, while maintaining a certain degree of experimental control over complex, reciprocal, and interactive influences applying a structured social interaction paradigm. Conducting equivalence testing to our data^55,56^, we show that a large effect of gaze anxiety on gaze behavior in natural daily social interactions is unlikely. While our findings do not permit us to definitively exclude a (small-to-medium) effect of this relationship, they do compellingly reveal a striking contrast between subjective (η^2^ = 0.933) and objective measures (all η^2^ < 0.017) of gaze behavior. This contrast reveals and adds credence to the role of cognitive biases and distorted self-perception in individuals with social anxiety^64–66^ and suggests that these individuals are able to behaviorally compensate for their anxiety in order to show norm-conforming eye-contact^31,32^. This assumption is backed up by the fact that both participants and their interaction partners rated the interaction similarly positively after the experiment.

From a translational perspective, our findings indicate that treating gaze-anxious individuals psychotherapeutically by focusing on the acquisition of new social skills—such as increasing eye contact^67^ may be insufficient. Rather, psychotherapists should consider therapeutic approaches that target cognitive distortions regarding performance in social situations^68^. As both groups of participants with high and low gaze anxiety rated the interaction similarly positive, it could also be a hint of self-reports of gaze avoidance resulting from distorted memories rather than immediate evaluation^69^. In cognitive behavioral therapy, detecting such distortions and replacing them by more realistic and functional beliefs (e.g. by identifying and restructuring automatically-negative thoughts^70^) is considered fundamental to the treatment process^71,72^. This concept of cognitive distortions as constructs that arise from inadequate information processing—such as catastrophizing, all-or-nothing thinking, and overgeneralizing—was first introduced in models on depression^73,74^. Cognitive models of SAD have adopted the idea of cognitive distortions and, indeed, one observed increased distortions in SAD patients as well^75^. Yet there is very little empirical evidence identifying cognitive distortions that are specific for anxiety^76^, which might be attributable to the fact that components of anxiety and depression are hard to disentangle given their high comorbidity^77^. In this regard, our study suggests that future endeavors to identify anxiety-specific distortions and corresponding treatment strategies should focus on dysfunctional beliefs about the adequacy of one’s eye-contact and social performance in general. Outpatient clinics with access to research facilities might also provide SAD patients with the opportunity for objectively quantifying and checking their beliefs regarding their gaze behavior by utilizing the dual-eye tracking setup described in this study.

Beyond providing specific knowledge on the relationship between gaze anxiety and gaze behavior, our study demonstrates the huge potential of dual eye-tracking in revealing interactive gaze behavior during naturalistic face-to-face interactions. While eye-tracking experiments studying responses to computer-based social stimuli have yielded valuable insights into how our eyes process social stimuli^25,78^, recent studies have suggested that gaze behavior in daily social interactions might differ starkly from gaze behavior towards static social stimuli^34–36^. These differences between experimental setups might partly be behind the inconsistent findings observed in previous studies regarding gaze anxiety’s effects on gaze behavior (for a recent review, see^79^). By studying gaze behavior during a naturalistic face-to-face interaction and analyzing both classical one-way and two-way interactive eye-tracking parameters, we demonstrate here that the effect of gaze anxiety on gaze behavior is smaller than expected from self-reports, even when we take the opponent’s gaze behavior into account. Our results show, that highly gaze-anxious individuals dwelled on their opponent’s eyes for a similar duration as individuals with low gaze anxiety, and they even did so when their opponent was dwelling on their eyes too, i.e. during mutual eye-contact. This is important additional information, because socially anxious individuals are more likely to perceive their opponent’s gaze as being directed towards them^9,80^, which they are most likely to find particularly aversive.

In sum, the current study delivers good news for individuals suffering from gaze anxiety: their gaze behavior might be much less compromised than they fear and, as indicated by the analyses on the subjective ratings of interaction quality, they seem able to interact in a functionally unobtrusive way despite their gaze anxiety. Future studies could include arousal-related physiological measures (e.g. heart rate or electrodermal activity)^31,81^ to see whether the normal gaze behavior observed in gaze-anxious individuals comes at any cost to them; a limitation we could not address with our study design. It could be that while interacting, these individuals find that they have to regulate their anxiety to fulfil the normative standard of normal gaze behavior exhibited from face-to-face. Perhaps, being able to give such individuals feedback from objective measures demonstrating that their own and their opponent’s actual gaze behaviors are largely unaffected by their high anxiety could help dispel someone’s fear of performing poorly in social situations. It could also prove worthwhile and informative to check whether our study’s findings apply to social settings involving explicitly negative social evaluations or conflicts^27^ and to individuals with clinically relevant levels of social anxiety. A critical issue that should also be addressed is whether gaze behavior displayed during the dual eye-tracking procedure generalizes to real world gaze behavior, given that the purpose of this eye-tracking study design is still not perfectly concealed and that the setup might still have made participants aware of the specific test situation. Overall, given the broad significance of eye-contact in enabling functional face-to-face social interactions, this study’s minimally invasive dual eye-tracking setup could prove to be of great utility for researchers across the social, clinical, and behavioral sciences.

## Supplementary Information

Supplementary Tables.

## Data Availability

Derived data supporting the findings of this study are available from the corresponding author on request.

## References

[1] Emery NJ (2000). The eyes have it: The neuroethology, function and evolution of social gaze. Neurosci. Biobehav. Rev..

[2] Langton SRH, Watt RJ, Bruce V (2000). Do the eyes have it? Cues to the direction of social attention. Trends Cogn. Sci..

[3] Bindemann M, Burton AM, Langton SRH (2008). How do eye gaze and facial expression interact?. Vis. Cogn..

[4] Senju A, Johnson MH (2009). The eye contact effect: Mechanisms and development. Trends Cogn. Sci..

[5] Piazza EA, Hasenfratz L, Hasson U, Lew-Williams C (2020). Infant and adult brains are coupled to the dynamics of natural communication. Psychol. Sci..

[6] Chen J, Short M, Kemps E (2020). Interpretation bias in social anxiety: A systematic review and meta-analysis. J. Affect. Disord..

[7] Bantin T, Stevens S, Gerlach AL, Hermann C (2016). What does the facial dot-probe task tell us about attentional processes in social anxiety? A systematic review. J. Behav. Ther. Exp. Psychiatry.

[8] Chen NTM, Clarke PJF (2017). Gaze-based assessments of vigilance and avoidance in social anxiety: A review. Curr. Psychiatry Rep..

[9] Schulze L, Lobmaier JS, Arnold M, Renneberg B (2013). All eyes on me?! Social anxiety and self-directed perception of eye gaze. Cogn. Emot..

[10] Auyeung B, Lombardo MV, Heinrichs M, Chakrabarti B, Sule A, Deakin JB, Baron-Cohen S (2015). Oxytocin increases eye contact during a real-time, naturalistic social interaction in males with and without autism. Transl. Psychiatry.

[11] Craske MG, Stein MB (2016). Anxiety. Lancet.

[12] Ruscio AM, Brown TA, Chiu WT, Sareen J, Stein MB, Kessler RC (2008). Social fears and social phobia in the USA: Results from the National comorbidity survey replication. Psychol. Med..

[13] Heimberg RG, Hofmann SG, Liebowitz MR, Schneier FR, Smits JA, Stein MB, Craske MG (2014). Social anxiety disorder in DSM-5. Depress. Anxiety.

[14] Baker A, Lewin T, Reichler H, Clancy R, Carr Garrett V, Terry M (2002). Evaluation of a motivational interview for substance use within psychiatric in-patient services. Addiction.

[15] Safren SA, Heimberg RG, Horner KJ, Juster HR, Schneier FR, Liebowitz MR (1999). Factor structure of social fears: The Liebowitz Social Anxiety Scale. J. Anxiety Disord..

[16] Schneier FR, Abi-Dargham A, Martinez D, Slifstein M, Hwang D-R, Liebowitz MR, Laruelle M (2009). Dopamine transporters, D2 receptors, and dopamine release in generalized social anxiety disorder. Depress. Anxiety.

[17] Horley K, Williams LM, Gonsalvez C, Gordon E (2004). Face to face: Visual scanpath evidence for abnormal processing of facial expressions in social phobia. Psychiatry Res..

[18] Moukheiber A, Rautureau G, Perez-Diaz F, Soussignan R, Dubal S, Jouvent R, Pelissolo A (2010). Gaze avoidance in social phobia: Objective measure and correlates. Behav. Res. Ther..

[19] Moukheiber A, Rautureau G, Perez-Diaz F, Jouvent R, Pelissolo A (2012). Gaze behaviour in social blushers. Psychiatry Res..

[20] Schneier FR, Rodebaugh TL, Blanco C, Lewin H, Liebowitz MR (2011). Fear and avoidance of eye contact in social anxiety disorder. Compr. Psychiatry.

[21] Greist JH (1995). The diagnosis of social phobia. J. Clin. Psychiatry.

[22] Öhman A (1986). Face the beast and fear the face: Animal and social fears as prototypes for evolutionary analyses of emotion. Psychophysiology.

[23] Domes G, Marx L, Spenthof I, Heinrichs M (2016). The German version of the Gaze Anxiety Rating Scale (GARS): Reliability and validity. PLoS ONE.

[24] Weeks JW, Howell AN, Srivastav A, Goldin PR (2019). “Fear guides the eyes of the beholder”: Assessing gaze avoidance in social anxiety disorder via covert eye tracking of dynamic social stimuli. J. Anxiety Disord..

[25] Boll S, Bartholomaeus M, Peter U, Lupke U, Gamer M (2016). Attentional mechanisms of social perception are biased in social phobia. J. Anxiety Disord..

[26] Hessels RS, Holleman GA, Cornelissen THW, Hooge ITC, Kemner C (2018). Eye contact takes two-autistic and social anxiety traits predict gaze behavior in dyadic interaction. J. Exp. Psychopathol..

[27] Langer JK, Lim MH, Fernandez KC, Rodebaugh TL (2017). Social anxiety disorder is associated with reduced eye contact during conversation primed for conflict. Cogn. Ther. Res..

[28] Mansour H, Kuhn G (2019). Studying “natural” eye movements in an “unnatural” social environment: The influence of social activity, framing, and sub-clinical traits on gaze aversion. Q. J. Exp. Psychol..

[29] Rogers SL, Speelman CP, Guidetti O, Longmuir M (2018). Using dual eye tracking to uncover personal gaze patterns during social interaction. Sci. Rep..

[30] Rubin M, Minns S, Muller K, Tong MH, Hayhoe MM, Telch MJ (2020). Avoidance of social threat: Evidence from eye movements during a public speaking challenge using 360°-video. Behav. Res. Ther..

[31] Rösler L, Göhring S, Strunz M, Gamer M (2021). Social anxiety is associated with heart rate but not gaze behavior in a real social interaction. J. Behav. Ther. Exp. Psychiatry.

[32] Wieser MJ, Pauli P, Alpers GW, Mühlberger A (2009). Is eye to eye contact really threatening and avoided in social anxiety?—An eye-tracking and psychophysiology study. J. Anxiety Disord..

[33] Vehlen A, Spenthof I, Tönsing D, Heinrichs M, Domes G (2021). Evaluation of an eye tracking setup for studying visual attention in face-to-face conversations. Sci. Rep..

[34] Laidlaw KEW, Foulsham T, Kuhn G, Kingstone A (2011). Potential social interactions are important to social attention. Proc. Natl. Acad. Sci..

[35] Macdonald RG, Tatler BW (2018). Gaze in a real-world social interaction: A dual eye-tracking study. Q. J. Exp. Psychol..

[36] Risko EF, Richardson DC, Kingstone A (2016). Breaking the fourth wall of cognitive science: Real-world social attention and the dual function of gaze. Curr. Dir. Psychol. Sci..

[37] Freeth M, Foulsham T, Kingstone A (2013). What affects social attention? Social presence, eye contact and autistic traits. PLoS ONE.

[38] Gobel MS, Kim HS, Richardson DC (2015). The dual function of social gaze. Cognition.

[39] Guerin B (1986). Mere presence effects in humans: A review. J. Exp. Soc. Psychol..

[40] Ho S, Foulsham T, Kingstone A (2015). Speaking and listening with the eyes: Gaze signaling during dyadic interactions. PLoS ONE.

[41] Tomasello M, Carpenter M (2007). Shared intentionality. Dev. Sci..

[42] Aron A, Melinat E, Aron EN, Vallone RD, Bator RJ (1997). The experimental generation of interpersonal closeness: A procedure and some preliminary findings. Pers. Soc. Psychol. Bull..

[43] Page-Gould E, Mendoza-Denton R, Tropp LR (2008). With a little help from my cross-group friend: Reducing anxiety in intergroup contexts through cross-group friendship. J. Pers. Soc. Psychol..

[44] Doherty-Sneddon G, Bruce V, Bonner L, Longbotham S, Doyle C (2002). Development of gaze aversion as disengagement from visual information. Dev. Psychol..

[45] Brustkern J, Heinrichs M, Walker M, Schiller B (2021). Facial threat affects trust more strongly than facial attractiveness in women than it does in men. Sci. Rep..

[46] Przybylski AK, Weinstein N (2013). Can you connect with me now? How the presence of mobile communication technology influences face-to-face conversation quality. J. Soc. Pers. Relat..

[47] Welker KM, Slatcher RB, Baker L, Aron A (2014). Creating positive out-group attitudes through intergroup couple friendships and implications for compassionate love. J. Soc. Pers. Relat..

[48] Stangier U, Heidenreich T, Berardi A, Golbs U, Hoyer J (1999). Die erfassung sozialer phobie durch social interaction anxiety scale (SIAS) und die social phobia scale (SPS). Z. Klin. Psychol..

[49] Mattick RP, Clarke JC (1998). Development and validation of measures of social phobia scrutiny fear and social interaction anxiety. Behav. Res. Therapy.

[50] Schmidt KH, Metzler P (1992). Wortschatztest [German Vocabulary Test].

[51] Aron A, Aron EN, Smollan D (1992). Inclusion of other in the self scale and the structure of interpersonal closeness. J. Pers. Soc. Psychol..

[52] Amos B, Ludwiczuk B, Satyanarayanan M (2016). Openface: A general-purpose face recognition library with mobile applications. CMU Sch. Comput. Sci..

[53] Hessels RS, Kemner C, van den Boomen C, Hooge ITC (2016). The area-of-interest problem in eyetracking research: A noise-robust solution for face and sparse stimuli. Behav. Res. Methods.

[54] Vehlen A, Standard W, Domes G (2022). How to choose the size of facial areas of interest in interactive eye tracking. PLoS ONE.

[55] IBM Corp. Released 2021. *IBM SPSS Statistics for Windows, Version 28.0*. (IBM Corp, 2021)

[56] Goertzen JR, Cribbie RA (2010). Detecting a lack of association: An equivalence testing approach. Br. J. Math. Stat. Psychol..

[57] Rogers JL, Howard KI, Vessey JT (1993). Using significance tests to evaluate equivalence between two experimental groups. Psychol. Bull..

[58] Lakens D, Scheel AM, Isager PM (2018). Equivalence testing for psychological research: A tutorial. Adv. Methods Pract. Psychol. Sci..

[59] Jeffreys H (1961). Theory of Probability.

[60] Rouder JN, Speckman PL, Sun D, Morey RD, Iverson G (2009). Bayesian t tests for accepting and rejecting the null hypothesis. Psychon. Bull. Rev..

[61] JASP Team. *JASP (Version 0.16) [Computer software]*. https://jasp-stats.org/ (2021).

[62] van Doorn J, van den Bergh D, Böhm U, Dablander F, Derks K, Draws T, Wagenmakers EJ (2021). The JASP guidelines for conducting and reporting a Bayesian analysis. Psychon. Bull. Rev..

[63] Konovalova I, Antolin JV, Bolderston H, Gregory NJ (2021). Adults with higher social anxiety show avoidant gaze behaviour in a real-world social setting: A mobile eye tracking study. PLoS ONE.

[64] Clark, D. M. A cognitive model. in *Social Phobia: Diagnosis, Assessment, and Treatment*. 69–73 (1995).

[65] Clark DM, McManus F (2002). Information processing in social phobia. Biol. Psychiat..

[66] Wong QJJ, Rapee RM (2016). The aetiology and maintenance of social anxiety disorder: A synthesis of complementary theoretical models and formulation of a new integrated model. J. Affect. Disord..

[67] Warner, C. M., Colognori, D., & Lynch, C. *Helping Students Overcome Social Anxiety: Skills for Academic and Social Success (SASS)*. (Guilford Publications, 2018).

[68] Craske MG, Niles AN, Burklund LJ, Wolitzky-Taylor KB, Vilardaga JCP, Arch JJ, Lieberman MD (2014). Randomized controlled trial of cognitive behavioral therapy and acceptance and commitment therapy for social phobia: Outcomes and moderators. J. Consult. Clin. Psychol..

[69] Mitte K (2008). Memory bias for threatening information in anxiety and anxiety disorders: A meta-analytic review. Psychol. Bull..

[70] Heimberg, R. G., & Becker, R. E. *Cognitive-Behavioral Group Therapy for Social Phobia: Basic Mechanisms and Clinical Strategies*. (Guilford Press, 2002).

[71] Butler RM, O’Day EB, Swee MB, Horenstein A, Heimberg RG (2021). Cognitive behavioral therapy for social anxiety disorder: Predictors of treatment outcome in a quasi-naturalistic setting. Behav. Ther..

[72] Flannery-Schroeder EC, Kendall PC (2000). Group and individual cognitive-behavioral treatments for youth with anxiety disorders: A randomized clinical trial. Cogn. Ther. Res..

[73] Beck AT (1979). Cognitive Therapy of Depression.

[74] Beck, J. S., & Beck, A. T. *Cognitive Therapy: Basics and Beyond*. (Guilford Press, 1995).

[75] Clark, D. A., & Beck, A. T. *Cognitive Therapy of Anxiety Disorders: Science and Practice*. (Guilford Press, 2011).

[76] Kuru E, Safak Y, Özdemir İ, Tulacı RG, Özdel K, Özkula NG, Örsel S (2018). Cognitive distortions in patients with social anxiety disorder: Comparison of a clinical group and healthy controls. Eur. J. Psychiatry.

[77] Hirschfeld RMA (2001). The comorbidity of major depression and anxiety disorders: Recognition and management in primary care. Prim. Care Companion J. Clin. Psychiatry.

[78] Arizpe J, Kravitz DJ, Walsh V, Yovel G, Baker CI (2016). Differences in looking at own-and other-race faces are subtle and analysis-dependent: An account of discrepant reports. PLoS ONE.

[79] Chen J, van den Bos E, Westenberg PM (2020). A systematic review of visual avoidance of faces in socially anxious individuals: Influence of severity, type of social situation, and development. J. Anxiety Disord..

[80] Gamer M, Hecht H, Seipp N, Hiller W (2011). Who is looking at me? The cone of gaze widens in social phobia. Cogn. Emot..

[81] Myllyneva A, Ranta K, Hietanen JK (2015). Psychophysiological responses to eye contact in adolescents with social anxiety disorder. Biol. Psychol..

