# Supplementary material for: No evidence that gaze anxiety predicts gaze avoidance behavior during face-to-face social interaction
Source: Sci Rep. 2022 Dec 9;12:21332. doi: 10.1038/s41598-022-25189-z (PMC9734162; doi:10.1038/s41598-022-25189-z)
Supplement: Supplementary file 1 — Supplementary Tables. [file 41598_2022_25189_MOESM1_ESM.docx]

**Gaze Anxiety Does Not Predict Gaze Avoidance Behavior during Face-to-Face Social Interaction**

Running head: Dyadic Dual Eye-Tracking During Social Interaction

Daniel Tönsing^1*^, Bastian Schiller^1,2*^, Antonia Vehlen^3^, Ines Spenthof^1^, Gregor Domes^3^ and Markus Heinrichs^1,2^

^1^Department of Psychology, Biological Psychology, Clinical Psychology and Psychotherapy, Albert-Ludwigs University of Freiburg, Freiburg, Germany

^2^Freiburg Brain Imaging Center, University Medical Center, Albert-Ludwigs University of Freiburg, Freiburg, Germany
^3^Department of Biological and Clinical Psychology, University of Trier, Trier, Germany

*these authors contributed equally

**Supplemental Material**

**Table S1.**

*Descriptive parameters of participant groups*

|  | **GARS: Low** | | **GARS: High** | |
| --- | --- | --- | --- | --- |
|  | *M* | *SD* | *M* | *SD* |
| **Sex** f (m) | n = 13 (14) | | n = 14 (10) | |
| **Age** | 23.88 | 3.30 | 24.63 | 4.18 |
| **IQ** | 104.67 | 4.60 | 102.29 | 8.66 |
| **GARS** | 6.58 | 3.01 | 39.17 | 5.65 |
| *GARS-Fear* | 2.19 | 2.10 | 19.38 | 4.24 |
| *GARS-Avoidance* | 4.44 | 1.99 | 22.63 | 4.55 |
| **SIAS** | 13.08 | 7.10 | 25.78 | 9.11 |

*Note.* GARS: Gaze Anxiety Rating Scale, SIAS: Social Interaction Anxiety Scale, IQ: Intelligent quotient

(measured by Verbal Intelligence Test). Quantity (n), Mean (*M*) and standard deviation (*SD*) are reported.

**Table S2.**

*Fast friends procedure: All twelve questions in German (applied) and original questions in English*

| 1. Wenn du dich mit irgendeinem Menschen auf der Welt zum Abendessen verabreden dürftest, wen würdest du wählen?   (*Given the choice of anyone in the world, whom would you want as a dinner guest?)*   1. Wärst du gerne berühmt? Wenn ja wofür?   *(Would you like to be famous? In what way*?)   1. Gehst du manchmal vor einem Telefonat durch, was du sagen möchtest? Warum?   *(Before making a telephone call, do you ever rehearse what you are going to say? Why?)*   1. Was würde einen perfekten Tag für dich ausmachen?   *(What would constitute a “perfect” day for you?)*   1. Wann hast du das letzte Mal für dich alleine gesungen? Wann vor jemand anderem?   (*When did you last sing to yourself? To someone else?)*   1. Wenn du in der Lage wärst, entweder den Geist oder den Körper eines 25-Jährigen dein ganzes Leben lang zu bewahren, was würdest du wählen und warum?   *(If you were able to live to the age of 90 and retain either the mind or body of a 30-year-old for the last 60 years of your life, which would you want?)*   1. Nenne drei Dinge, die du und dein Gesprächspartner gemeinsam zu haben scheinen.   *(Name three things you and your partner appear to have in common.)*   1. Wenn du morgen mit einer neuen Eigenschaft oder Fähigkeit deiner Wahl aufwachen könntest, welche wäre das? Warum?   *(If you could wake up tomorrow having gained any one quality or ability, what would it be?)*   1. Für was in deinem Leben bist du am dankbarsten? Warum?   *(For what in your life do you feel most grateful?*   1. Wenn du etwas daran ändern könntest, wie du aufgewachsen bist, was wäre das und warum?   *(If you could change anything about the way you were raised, what would it be?)*   1. Wenn eine Kristallkugel dir die Wahrheit über dich, dein Leben, die Zukunft oder irgendetwas anderes verraten könnte, was würdest du wissen wollen? Warum?   (*If a crystal ball could tell you the truth about yourself, your life, the future, or anything else, what would you want to know?)*   1. Nimm dir drei Minuten Zeit und erzähle deinem Partner deine Lebensgeschichte.   (*Take four minutes and tell your partner your life story in as much detail as possible*) |
| --- |

*Note.* Original questions in English are displayed in brackets

**Table S3.**

*Statistical analysis using a mixed ANOVA to compare participants in the “GARS low” and “GARS high” group (between-subjects factor “group”) in their two-way gaze behavior, “activity” (speaking vs. listening), and “sex” (female, male) for all Areas Of Interest (AOIs). Furthermore, non-parametric testing parameters are reported. Furthermore, we performed equivalence tests for each AOI to proof whether differences across GARS groups are smaller than what is considered to be a meaningful effect size in this population.*

|  |  | **Mixed ANOVA** | | | | **Non-Parametric Testing** | | | **Equivalence Test** | | | **Bayesian Testing** | |
| --- | --- | --- | --- | --- | --- | --- | --- | --- | --- | --- | --- | --- | --- |
| *AOI* |  | *df_Num_ & df_Den_* | *F* | *p* | *η^2^* | | *Z* | *p* | *df* | *T* | *p* | | *BF_01_* |
| **Eyes** | *GARS (low, high)* | 1, 51 | 0.07 | .796 | < .001 | | - 0.40 | .692 | 48.34 | 2.60 | .006 | | 3.45 |
|  | *Activity (speak., list.)* | 1, 51 | 3.90 | .051 | .040 | | - 1.09 | .275 | 49.31 | - 2.11 | .019 | |  |
|  | *Sex (female, male)* | 1, 51 | 3.42 | .068 | .035 | | - 1.51 | .131 | 47.46 | 1.37 | .088 | |  |
|  | *GARS x Activity* | 1, 51 | 0.73 | .394 | .008 | |  |  |  |  |  | |  |
|  | *GARS x Sex* | 1, 51 | 1.37 | .245 | .014 | |  |  |  |  |  | |  |
|  | *Activity x Sex* | 1, 51 | 0.30 | .587 | .003 | |  |  |  |  |  | |  |
|  | *GARS x Activity x Sex* | 1, 51 | 0.05 | .828 | <.001 | |  |  |  |  |  | |  |
| **Nose** | *GARS (low, high)* | 1, 51 | 0.34 | .564 | .003 | | - 0.70 | .485 | 42.02 | - 2.45 | .009 | | 3.23 |
|  | *Activity (speak., list.)* | 1, 51 | 15.78 | <.001 | .076 | | - 1.82 | .690 | 48.88 | - 2.29 | .012 | |  |
|  | *Sex (female, male)* | 1, 51 | 0.06 | .972 | .013 | | - 0.06 | .955 | 43.31 | - 2.67 | .005 | |  |
|  | *GARS x Activity* | 1, 51 | 0.05 | .821 | .001 | |  |  |  |  |  | |  |
|  | *GARS x Sex* | 1, 51 | 1.21 | .275 | .013 | |  |  |  |  |  | |  |
|  | *Activity x Sex* | 1, 51 | 0.08 | .777 | .001 | |  |  |  |  |  | |  |
|  | *GARS x Activity x Sex* | 1, 51 | .06 | .815 | .001 | |  |  |  |  |  | |  |
| **Mouth** | *GARS (low, high)* | 1, 51 | 0.02 | .881 | < .001 | | - 0.28 | .777 | 49.88 | -2.86 | .003 | | 3.56 |
|  | *Activity (speak., list.)* | 1, 51 | 4.73 | .032 | .048 | | - 2.18 | .029 | 47.33 | - 1.80 | .038 | |  |
|  | *Sex (female, male)* | 1, 51 | 1.95 | .166 | .020 | | - 1.47 | .141 | 48.64 | 1.78 | .040 | |  |
|  | *GARS x Activity* | 1, 51 | 0.04 | .836 | < .001 | |  |  |  |  |  | |  |
|  | *GARS x Sex* | 1, 51 | <0.01 | .980 | < .001 | |  |  |  |  |  | |  |
|  | *Activity x Sex* | 1, 51 | <0.01 | .954 | < .001 | |  |  |  |  |  | |  |
|  | *GARS x Activity x Sex* | 1, 51 | 0.04 | .847 | < .001 | |  |  |  |  |  | |  |
| **Face** | *GARS (low, high)* | 1, 51 | 0.12 | .773 | .001 | | 0.00 | .999 | 49.68 | 2.56 | .007 | | 4.42 |
|  | *Activity (speak., list.)* | 1, 51 | 10.31 | .002 | .099 | | - 1.13 | .258 | 45.16 | - 0.97 | .167 | |  |
|  | *Sex (female, male)* | 1, 51 | 5.31 | .023 | .053 | | - 1.77 | .076 | 39.68 | 0.76 | .221 | |  |
|  | *GARS x Activity* | 1, 51 | 0.83 | .366 | .009 | |  |  |  |  |  | |  |
|  | *GARS x Sex* | 1, 51 | 8.42 | .005 | .082 | |  |  |  |  |  | |  |
|  | *Activity x Sex* | 1, 51 | 0.45 | .506 | .005 | |  |  |  |  |  | |  |
|  | *GARS x Activity x Sex* | 1, 51 | 0.90 | .344 | .010 | |  |  |  |  |  | |  |

*Note. AOI: Areas of interest; df_Num_* indicates degrees of freedom numerator*. df_Den_* indicates degrees of freedom denominator. *η^2^* indicates partial eta squared. *BF_01_* Bayesian Factor giving the evidence for H_0_ over H_1_

**Table S4.**

*Statistical analysis using a mixed ANOVA to compare the “GARS low” and “GARS high” group (between-subjects factor “group”) in their two-way gaze behavior, “activity” (speaking vs. listening), and “sex” (female, male) for mutual eye and mutual face gaze. Furthermore, non-parametric testing is reported. Equivalence tests test whether differences across GARS groups are smaller than meaningful effect size in this population.*

|  |  | **Mixed ANOVA** | | | | **Non-Parametric Testing** | | | **Equivalence Test** | | | **Bayesian Testing** | |
| --- | --- | --- | --- | --- | --- | --- | --- | --- | --- | --- | --- | --- | --- |
| *Varibale* | | *df_Num_ & df_Den_* | *F* | *p* | *η^2^* | | *Z* | *p* | *df* | *T* | *p* | | *BF_01_* |
| **Mutual Eye Gaze** | *GARS (low, high)* | 1, 51 | 0.01 | .920 | < .001 | | -0.21 | .834 | 46.90 | 2.71 | .005 | | 3.43 |
|  | *Activity (speak., list.)* | 1, 51 | 0.65 | .423 | .007 | | -1.34 | .180 | 41.66 | 3.01 | .002 | |  |
|  | *Sex (female, male)* | 1, 51 | 0.27 | .546 | .004 | | -0.39 | .696 | 43.23 | 3.24 | < .001 | |  |
|  | *GARS x Activity* | 1, 51 | 0.03 | .861 | < .001 | |  |  |  |  |  | |  |
|  | *GARS x Sex* | 1, 51 | 0.76 | .386 | .008 | |  |  |  |  |  | |  |
|  | *Activity x Sex* | 1, 51 | 0.33 | .548 | .004 | |  |  |  |  |  | |  |
|  | *GARS x Activity x Sex* | 1, 51 | 0.02 | .889 | < .001 | |  |  |  |  |  | |  |
| **Mutual Face Gaze** | *GARS (low, high)* | 1, 51 | 0.23 | .635 | .002 | | 0.56 | .573 | 48.78 | 3.61 | < .001 | | 3.45 |
|  | *Activity (speak., list.)* | 1, 51 | 0.01 | .909 | < .001 | | -1.01 | .311 | 48.92 | - 4.08 | < .001 | |  |
|  | *Sex (female, male)* | 1, 51 | 0.02 | .885 | < .001 | | -0.72 | .472 | 42.36 | - 3.96 | < .001 | |  |
|  | *GARS x Activity* | 1, 51 | 1.02 | .319 | .011 | |  |  |  |  |  | |  |
|  | *GARS x Sex* | 1, 51 | 0.80 | .373 | .008 | |  |  |  |  |  | |  |
|  | *Activity x Sex* | 1, 51 | 0.91 | .342 | .010 | |  |  |  |  |  | |  |
|  | *GARS x Activity x Sex* | 1, 51 | 0.15 | .694 | .002 | |  |  |  |  |  | |  |

*Note. df_Num_* indicates degrees of freedom numerator*. df_Den_* indicates degrees of freedom denominator. *η^2^* indicates partial eta squared, Gaze Anxiety Rating Scale (GARS). *BF_01_* Bayesian Factor giving the evidence for H_0_ over H_1_

**Table S5**

*Correlations analyses to check the coherencies between subjective gaze anxiety and eye tracking data for group “GARS low”*

|  |  |  | **One-way Gaze Data** | | | | **Two-way Gaze Data** | |
| --- | --- | --- | --- | --- | --- | --- | --- | --- |
| Group | Subscale |  | Dwell Time on Eyes | Dwell Time on Face | Mean Fixation Time on Eyes | Mean Fixation Time on Face | Mutual Eye Gaze | Mutual Face Gaze |
| **GARS** | *GARS* | *rp* | 0.101 | 0.317 | 0.347 | 0.238 | 0.323 | 0.419 |
| **low** |  | *p* | .615 | .108 | .076 | .232 | .101 | .029* |
|  |  | *rs* | 0.082 | 0.295 | 0.230 | 0.234 | 0.322 | 0.428 |
|  |  | *p* | .683 | .136 | .249 | .240 | .101 | .026* |
|  | *GARS - Avoidance* | *rp* | - 0.028 | 0.461 | 0.349 | 0.360 | 0.301 | 0.485 |
|  |  | *p* | .889 | .016* | .075 | .065 | .128 | .010* |
|  |  | *rs* | -0.009 | 0.448 | 0.288 | 0.334 | 0.330 | 0.526 |
|  |  | *p* | .963 | .019* | .146 | .089 | .093 | .005* |
|  | *GARS - Fear* | *rp* | 0.170 | 0.011 | 0.160 | -0.004 | 0.171 | 0.133 |
|  |  | *p* | .397 | .957 | .424 | .982 | .393 | .509 |
|  |  | *rs* | 0.172 | 0.058 | 0.109 | 0.074 | 0.184 | 0.160 |
|  |  | *p* | .392 | .775 | .588 | .715 | .359 | .424 |

*Note.* Regression coefficients for Person (*rp*) and Spearman rang correlation (*rs*) for individuals reporting low levels of gaze anxiety (GARS low). Gaze Anxiety Rating Scale (GARS)

**Table S6**

*Correlations analyses to check the coherencies between subjective gaze anxiety and eye tracking data for group “GARS high”*

|  |  |  | **One-way Gaze Data** | | | | **Two-way Gaze Data** | |
| --- | --- | --- | --- | --- | --- | --- | --- | --- |
| Group | Subscale |  | Dwell Time on Eyes | Dwell Time on Face | Mean Fixation Time on Eyes | Mean Fixation Time on Face | Mutual Eye Gaze | Mutual Face Gaze |
| **GARS** | *GARS* | *rp* | -0.027 | -0.159 | -0.151 | -0.088 | -0.346 | -0.133 |
| **high** |  | *p* | .900 | .458 | .482 | .684 | .098 | .535 |
|  |  | *rs* | -0.071 | -0.161 | -0.053 | -0.122 | -0.322 | -0.148 |
|  |  | *p* | .741 | .452 | .806 | .569 | .125 | .490 |
|  | *GARS - Avoidance* | *rp* | 0.163 | -0.091 | -0.076 | -0.166 | -0.264 | -0.126 |
|  |  | *p* | .447 | .672 | .725 | .439 | .213 | .559 |
|  |  | *rs* | -0.020 | -0.136 | 0.044 | -0.097 | -0.228 | -0.205 |
|  |  | *p* | .925 | .527 | .840 | .651 | .283 | .337 |
|  | *GARS - Fear* | *rp* | -0.115 | -0.231 | -0.254 | -0.140 | -0.273 | -0.111 |
|  |  | *p* | .594 | .276 | .231 | .514 | .197 | .607 |
|  |  | *rs* | -0.124 | -0.102 | -0.014 | 0.039 | -0.287 | -0.112 |
|  |  | *p* | .562 | .634 | .948 | .855 | .173 | .603 |

*Note.* Regression coefficients for Person (*rp*) and Spearman rang correlation (*rs*), for individuals reporting high levels of gaze anxiety (GARS high). Gaze Anxiety Rating Scale (GARS)

**Table S7**

*Correlations analyses to check the coherencies between subjective gaze anxiety and eye tracking data for group “GARS high”*

|  |  |  | **One-way Gaze Data** | | | | **Two-way Gaze Data** | |
| --- | --- | --- | --- | --- | --- | --- | --- | --- |
| Group | Scale |  | Dwell Time on Eyes | Dwell Time on Face | Mean Fixation Time on Eyes | Mean Fixation Time on Face | Mutual Eye Gaze | Mutual Face Gaze |
| **GARS low** | SIAS | *rp* | 0.063 | 0.341 | 0.352 | 0.384 | 0.058 | 0.195 |
|  |  | *p* | .755 | .082 | .072 | .048* | .772 | .330 |
|  |  | *rs* | 0.025 | 0.335 | 0.143 | 0.340 | - 0.018 | 0.104 |
|  |  | *p* | .900 | .087 | .478 | .083 | .929 | .607 |
| **GARS high** | SIAS | *rp* | -0.106 | -0.247 | -0.145 | -0.196 | -0.272 | -0.295 |
|  |  | *p* | .620 | .244 | .499 | .358 | .199 | .161 |
|  |  | *rs* | -0.059 | -0.209 | -0.222 | -0.277 | -0.183 | -0.272 |
|  |  | *p* | .785 | .327 | .297 | .191 | .391 | .199 |

*Note.* Pearson correlation coefficients (*rp*), Spearman rank correlation (*rs*), for individuals reporting low levels of gaze anxiety (GARS low) and individuals report high levels of gaze anxiety(GARS high). Gaze Anxiety Rating Scale (GARS)

**Table S8.**

*Subjective ratings of interaction quality for both groups*

|  | GARS low | | GARS high | | | Parametric Testing | | | | Non-Parametric Testing | | |  |
| --- | --- | --- | --- | --- | --- | --- | --- | --- | --- | --- | --- | --- | --- |
|  | *M* | *SD* | | *M* | *SD* | | *t(*51*)* | *p* | *d* | | *Z* | *p* | |
| Inclusion of Other | 4.15 | 1.29 | | 4.29 | 1.55 | | 0.36 | .360 | 0.10 | | -0.25 | .800 | |
| Liking | 5.81 | 0.83 | | 5.79 | 0.72 | | 0.11 | .458 | 0.03 | | -0.16 | .871 | |
| General Feeling | 5.63 | 1.01 | | 5.75 | 0.85 | | 0.46 | .324 | 0.13 | | 0.32 | .747 | |
| Interested to interact again | 6.19 | 1.00 | | 6.21 | 1.14 | | 0.08 | .469 | 0.02 | | 0.20 | .845 | |
| Similarity | 5.11 | 1.12 | | 5.25 | 1.07 | | 0.45 | .327 | 0.13 | | 0.61 | .545 | |
| Enjoyment | 5.59 | 0.97 | | 5.38 | 0.77 | | 0.88 | .192 | 0.24 | | -0.77 | .443 | |
| Laugh | 5.52 | 1.12 | | 5.50 | 1.25 | | 0.06 | .478 | 0.02 | | -0.01 | .992 | |
| Fun | 5.74 | 0.98 | | 5.54 | 0.88 | | 0.76 | .227 | 0.21 | | -0.81 | .419 | |
| Self-revelation | 5.70 | 1.03 | | 5.46 | 0.59 | | 1.03 | .155 | 0.28 | | -1.56 | .120 | |
| Intimate information | 5.07 | 1.27 | | 4.63 | 0.97 | | 1.43 | .800 | 0.39 | | -1.71 | .088 | |
| Honesty | 6.19 | 0.88 | | 6.04 | 0.69 | | 0.65 | .517 | 0.19 | | -0.89 | .371 | |
| SCI 1 | 4.33 | 1.78 | | 4.33 | 2.10 | | 0.00 | .500 | < 0.01 | | 0.13 | .893 | |
| SCI 2 | 4.41 | 1.65 | | 4.67 | 2.06 | | 0.50 | .310 | 0.14 | | 0.58 | .565 | |

*Note.* Means (*M*), standard deviations (*SD*), t-test results (*t*), and effect size (Cohen’s d) for individuals reporting high levels of gaze anxiety (GARS high) and individuals report low levels of gaze anxiety. Gaze Anxiety Rating Scale (GARS)

**Table S9**

*Subjective ratings of interaction quality for group’s interaction partners*

|  | GARS low | | GARS high | | | Parametric Testing | | | Non-Parametric Testing | | |  |
| --- | --- | --- | --- | --- | --- | --- | --- | --- | --- | --- | --- | --- |
|  | *M* | *SD* | | *M* | *SD* | *t(51)* | *p* | *d* | | *Z* | *p* | |
| Inclusion of Other | 4.30 | 1.33 | | 4.42 | 1.25 | 0.33 | .370 | 0.09 | | -0.46 | .646 | |
| Liking | 5.52 | 0.64 | | 5.75 | 0.79 | 1.15 | .128 | 0.32 | | -1.23 | .218 | |
| General Feeling | 5.63 | 0.93 | | 5.63 | 1.01 | 0.02 | .493 | < 0.01 | | 0.14 | .885 | |
| Interested to interact again | 5.93 | 1.19 | | 6.00 | 0.83 | 0.13 | .450 | 0.07 | | 0.39 | .697 | |
| Similarity | 4.89 | 0.97 | | 5.17 | 1.20 | 0.91 | .184 | 0.26 | | -0.93 | .354 | |
| Enjoyment | 5.33 | 1.24 | | 5.50 | 0.98 | 0.53 | .300 | 0.15 | | -0.33 | .744 | |
| Laugh | 5.22 | 1.25 | | 5.21 | 1.22 | 0.04 | .484 | 0.01 | | 0.20 | .845 | |
| Fun | 5.59 | 0.93 | | 5.63 | 1.06 | 0.12 | .454 | 0.04 | | -0.22 | .827 | |
| Self-revelation | 5.52 | 1.06 | | 5.54 | 0.70 | 0.10 | .461 | 0.02 | | -0.32 | .750 | |
| Intimate information | 4.93 | 1.24 | | 4.71 | 1.37 | 0.60 | .277 | 0.17 | | -0.40 | .690 | |
| Honesty | 6.07 | 0.92 | | 6.17 | 0.87 | 0.37 | .357 | 0.11 | | -0.36 | .722 | |
| Relationship (in comp. to friends) | 4.59 | 1.67 | | 4.75 | 1.42 | 0.36 | .360 | 0.10 | | -0.18 | .855 | |
| Relationship (in general) | 4.85 | 1.75 | | 4.63 | 1.17 | 0.54 | .297 | 0.15 | | 0.72 | .475 | |

*Note.* Means (*M*), standard deviations (*SD*), t-test results (*t*), and effect size (Cohen’s d) for individuals reporting high levels of gaze anxiety (GARS high) and individuals report low levels of gaze anxiety. Gaze Anxiety Rating Scale (GARS)
